# Intensive Care Unit Admission Parameters Improve the Accuracy of Operative Mortality Predictive Models in Cardiac Surgery

**DOI:** 10.1371/journal.pone.0013551

**Published:** 2010-10-21

**Authors:** Marco Ranucci, Andrea Ballotta, Serenella Castelvecchio, Ekaterina Baryshnikova, Simonetta Brozzi, Alessandra Boncilli, for the Surgical and Clinical Outcome Research (SCORE) Group

**Affiliations:** Department of Cardiothoracic-Vascular Anesthesia and Intensive Care, IRCCS Policlinico San Donato, San Donato Milanese, Italy; University of Chicago, United States of America

## Abstract

**Background:**

Operative mortality risk in cardiac surgery is usually assessed using preoperative risk models. However, intraoperative factors may change the risk profile of the patients, and parameters at the admission in the intensive care unit may be relevant in determining the operative mortality. This study investigates the association between a number of parameters at the admission in the intensive care unit and the operative mortality, and verifies the hypothesis that including these parameters into the preoperative risk models may increase the accuracy of prediction of the operative mortality.

**Methodology:**

929 adult patients who underwent cardiac surgery were admitted to the study. The preoperative risk profile was assessed using the logistic EuroSCORE and the ACEF score. A number of parameters recorded at the admission in the intensive care unit were explored for univariate and multivariable association with the operative mortality.

**Principal Findings:**

A heart rate higher than 120 beats per minute and a blood lactate value higher than 4 mmol/L at the admission in the intensive care unit were independent predictors of operative mortality, with odds ratio of 6.7 and 13.4 respectively. Including these parameters into the logistic EuroSCORE and the ACEF score increased their accuracy (area under the curve 0.85 to 0.88 for the logistic EuroSCORE and 0.81 to 0.86 for the ACEF score).

**Conclusions:**

A double-stage assessment of operative mortality risk provides a higher accuracy of the prediction. Elevated blood lactates and tachycardia reflect a condition of inadequate cardiac output. Their inclusion in the assessment of the severity of the clinical conditions after cardiac surgery may offer a useful tool to introduce more sophisticated hemodynamic monitoring techniques. Comparison between the predicted operative mortality risk before and after the operation may offer an assessment of the operative performance.

## Introduction

Risk stratification for operative mortality after cardiac operations in adult patients may be achieved using different risk scores [1]–[6]. Usually developed using logistic regression analyses, with the inclusion of different kinds and number of variables (ranging from 3 to 17), they all share the strategy of considering only preoperative or procedural risk factors.

Studies focused on hemodynamic, respiratory and metabolic parameters at the admission in the intensive care unit (ICU) following cardiac operations have demonstrated that some of these parameters are associated with postoperative morbidity and mortality. Early postoperative hyperlactatemia was identified as an independent predictor of morbidity and mortality after cardiac operations [7]. Other authors have tried to develop risk models based on ICU admission parameters in cardiac surgery. Higgins and coworkers [8], in a population of patients who undergone coronary revascularization, developed a model based on preoperative (age, history of preoperative vascular disease or interventions, serum albumin value), intraoperative (cardiopulmonary bypass duration), and early postoperative (arterial bicarbonate, heart rate, use of intra-aortic balloon pump, and cardiac index) parameters. The main limitation of this study are the use of parameters that are not routinely measured after cardiac surgery (cardiac index), the lack of potentially important parameters (blood lactates) that were not routinely measured at that time, and the inclusion of therapeutic measures (intra-aortic balloon pump) that are not patient-based signals.

General severity systems like Acute Physiology and Chronic Health Evaluation (APACHE), Simplified Acute Physiology Score (SAPS), and Mortality Probability Models (MPM), when applied to cardiac surgical patients, do not perform very well in predicting hospital mortality, and their accuracy is lower than the one of the preoperative Parsonnet model [9].

At present, there is no information about the value of routine parameters at the admission in the ICU in changing the accuracy of operative risk predictors in adult cardiac surgery.

The present study is aimed to (i) finding associations between routine parameters at the admission in the ICU and operative mortality following cardiac operations, and to (ii) verifying if the inclusion of these parameters improves the accuracy of the standard preoperative risk models in predicting operative mortality.

## Methods

This is a retrospective analysis of data prospectively collected in our institutional database. The Local Ethics Committee (ASL Milano 2) approved the study and waived the need for an informed consent of the patients, who however gave their written consent to the storage in the hospital database for scientific treatment of their data in an anonymous form at the time of the Hospital admission, according to the Italian law regulating personal privacy matters.

The cardiac surgical activity done in the year 2009 was reviewed. A total of 1,730 patients were analyzed. Pediatric patients (age <18 years), adult patients with congenital heart disease, and patients receiving cardiac procedures without cardiopulmonary bypass (CPB) were excluded. Patients missing a complete set of data at the admission in the ICU were excluded.

All the patients had a complete preoperative risk profile, and relevant intraoperative data were available. The preoperative risk for operative mortality was assessed using the logistic EuroSCORE [5] and the recently published ACEF score [6], which considers only age, creatinine and ejection fraction for stratifying the operative mortality risk in elective patients.

Data collected immediately at the admission in the ICU after cardiac operations and considered as potential predictors of operative mortality (mortality during the Hospital stay or within 30 days from the operation after discharge) were: arterial blood gas analysis parameters (pH, pCO_2_, HCO_3_^−^); arterial blood lactates (BL, mmol/L); hematocrit (%); mean arterial pressure (MAP, mmHg); heart rate (HR, beats per minute); central venous pressure (CVP, mmHg); core temperature (°C); arterial oxygen pressure to inspiratory oxygen fraction ratio (PaO_2_/FiO_2_). In case of cardiac pacing due to atrio-ventricular block or low-frequency sinus rhythm, the heart rate was measured with the pace-maker off (excepted in cases of complete atrioventricular block). Atrio-ventricular block was considered within the possible factors determining operative mortality.

A limited number of 52 patients had a pulmonary artery catheter positioned, and therefore hemodynamic parameters like cardiac index, mixed venous oxygen saturation, and related measures were not considered in this analysis.

Parameters depending on therapeutic measures (use of inotropic drugs; use of mechanical assist devices) were not considered in this analysis.

Data at the admission in the ICU were explored for univariate association with operative mortality using a Mann-Whitney U-test. The parameters being associated with operative mortality at a P value <0.1 were considered potentially includable in a multivariable analysis (stepwise forward logistic regression). Adequate measures of intercorrelation were applied, and to avoid collinearity and overfitting of the model a limited number of variables was admitted to the multivariable model.

The linearity assumption for the association between parameters at the admission in the ICU and operative mortality was tested by stratification of the patient population into relevant risk categories for the parameters being independent predictors of operative mortality. In case of non-linearity, adequate cut-off values were identified and tested for sensitivity, specificity, positive and negative predictive power. The parameters needing dichotomization were re-entered into a multivariable logistic regression analysis, and their weight was expressed in terms of an odds ratio (OR) with 95% confidence interval.

The preoperative risk models (logistic EuroSCORE and ACEF score) were tested for accuracy and calibration in predicting the operative mortality using a receiver operating characteristics (ROC) analysis, with the area under the curve (AUC) as a measure for accuracy and the Hosmer-Lemeshow statistics for testing calibration. The predicted and observed mortality rates were compared considering their 95% confidence interval.

Both the preoperative predictive models were re-adjusted by including the parameters at the admission in the ICU, whose weight was assessed based on their ORs. After this adjustment, the two models were re-tested for accuracy and calibration to investigate if the inclusion of the parameters at the admission in the ICU improved their predictive value.

Data are expressed as mean and standard deviation of the mean or aspercentage with 95% confidence interval when appropriate. For all the statistical tests a P value <0.05 was considered significant. All the statistical calculations were performed using a computerized statistical package (SPSS, Chicago, IL).

## Results

During the year 2009, 1,255 adult patients have been operated at our cardiac surgery center. Complete hemodynamic, respiratory and metabolic data at the admission in the ICU were available in our files for 929 patients.

There were 22 (2.5%, 95% confidence interval 1.4%–3.5%) operative deaths. Eighteen patients died due to a drug-refractory low cardiac output (complicated by sepsis and multi-organ failure in 4 patients and by respiratory failure in 2 patients). Three patients died due to perioperative stroke and 1 due to mediastinitis and subsequent sepsis with multi-organ failure.

Preoperative profile and intraoperative details of the patient population are depicted in Table 1. Patients suffering operative mortality had a significantly higher risk profile, characterized by an older age, lower left ventricular ejection fraction, higher serum creatinine level, and a higher rate of emergency operations (defined as operation to be performed within the working day). As a result, both the logistic EuroSCORE and the ACEF score were significantly higher in non-survivors. Moreover, they had a longer CPB duration, a lower CPB temperature, and a higher degree of hemodilution during CPB.

**Table 1:** Patient population profile (survivors and non-survivors).

Factor	Survivors	Non survivors	P
	(N = 907)	(N = 22)	
Age (years)	65.3±13	74.8±11	0.001
Gender male	619 (68)	13 (59)	0.362
Left ventricular ejection fraction (%)	54.5±12	44.3±13	0.001
Serum creatinine (mg/dL)	1.12±0.9	2.17±2.1	0.001
Hematocrit (%)	39.2±4.8	37.6±3.6	0.242
Recent myocardial infarction	84 (9)	2 (9)	0.999
Unstable angina	30 (3)	2 (9)	0.173
Chronic obstructive pulmonary disease	47 (5)	2 (9)	0.325
Diabetes on medication	144 (16)	3 (14)	0.985
Previous cerebrovascular accident	27 (3)	1 (5)	0.362
Redo operation	67 (7)	3 (14)	0.227
Emergent surgery	25 (3)	5 (23)	0.001
Isolated coronary operation	372 (41)	7 (32)	0.511
Cardiopulmonary bypass duration (min)	82±34	137±90	0.009
Aortic cross-clamping time (min)	58±27	86±42	0.005
Lowest temperature on CPB (°C)	32±1.6	31.2±2.4	0.021
Hypothermia (<25°C)	7 (0.7)	0 (0)	0.679
Lowest hematocrit on CPB (%)	26.3±3.5	24.1±3.7	0.005
Severe hemodilution (hematocrit <20%)	22 (2.4)	0 (0)	0.460
Intraoperative atrio-ventricular block	102 (11)	1 (4.7)	0.323
ACEF score	1.34±0.7	2.29±1.1	0.001
Logistic EuroSCORE	7.1±7.7	23.1±20.5	0.001

Data are expressed as mean ± standard deviation of the mean or number (%). P values according to Mann-Whitney U test. CPB: cardiopulmonary bypass.

Table 2 reports the parameters at the admission in the ICU. Six parameters were different between survivors and non-survivors at the pre-defined level of P<0.1. Non-survivors had a lower pH, a lower HCO_3_^−^ value, a higher pCO_2_, a higher BL value, a higher HR, a lower MAP and a higher CVP.

**Table 2:** Parameters at the admission in the Intensive Care Unit in survivors and non-survivors.

Factor	Survivors	Non survivors	P
	(N = 907)	(N = 22)	
PaO_2_/FiO_2_	308±177	295±196	0.359
pH	7.47±0.07	7.38±0.1	0.001
PaCO_2_ (mmHg)	35.5±5.8	40±7.8	0.001
HCO_3_^−^	25.8±3.2	24.3±2.7	0.006
Central temperature (°C)	36.1±1.6	35.4±2.3	0.513
Blood lactates (mmol/L)	1.36±0.81	2.95±3.5	0.042
Heart rate (beats/min)	85±16	97±20	0.002
Mean arterial pressure (mmHg)	99±19	89±22	0.047
Central venous pressure (mmHg)	8.7±3.6	10±3.5	0.061
Hematocrit (%)	30.9±4.1	30.2±4.4	0.359

Data are expressed as mean ± standard deviation. P values according to Mann-Whitney U test.

To avoid overfitting of the model, a limited number of the above factors was included in the multivariable analysis. pH, HCO_3_^−^, and pCO_2_ are mathematically coupled by the Henderson-Hasselbach equation, and only the pH value was therefore included. BL was intercorrelated with lower values of pH (P = 0.001) and HCO_3_^−^ (P = 0.04), and higher values of PCO_2_ (P = 0.001). HR, BL and MAP were included in the model, whereas CVP was excluded. The final multivariable (stepwise forward) logistic regression model was tested on these 4 parameters (table 3). Only HR and BL remained independent predictors of operative mortality. These two parameters were investigated for linearity relationship with operative mortality, and possible adequate cut-off values were searched.

**Table 3:** Independent predictors of operative mortality at the admission in the Intensive Care Unit.

Continuous variables				
*Factor*	*b coefficient*	*Odds Ratio*	*95% C.I.*	*P*
Blood lactates (mmol/L)	0.451	1.572	1.27–1.93	0.001
Heart rate (beats/min)	0.032	1.033	1.01–1.05	0.001
Constant	−7.45			

Dichotomous variables				
*Factor*	*b coefficient*	*Odds Ratio*	*95% C.I.*	*P*
Blood lactates >4 mmol/L	2.59	13.4	4.2–42	0.001
Heart rate >120 beats/min	1.91	6.7	1.6–28	0.011
Constant	−4.1			

**Figure 1:**
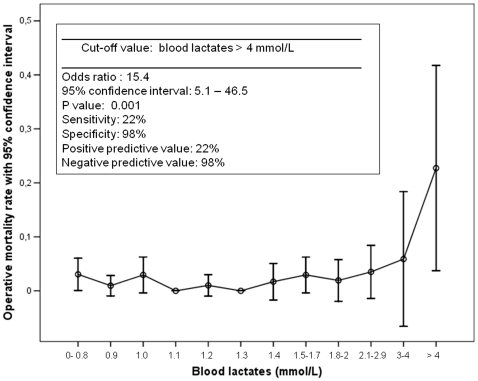
Blood lactates and operative mortality. Association between blood lactates at the admission in the intensive care unit for relevant categories, with parameters for the identified cut-off value.

Multivariable logistic regression analysis (stepwise forward). Variables dichotomized according to the values in figure 1.

**Figure 2:**
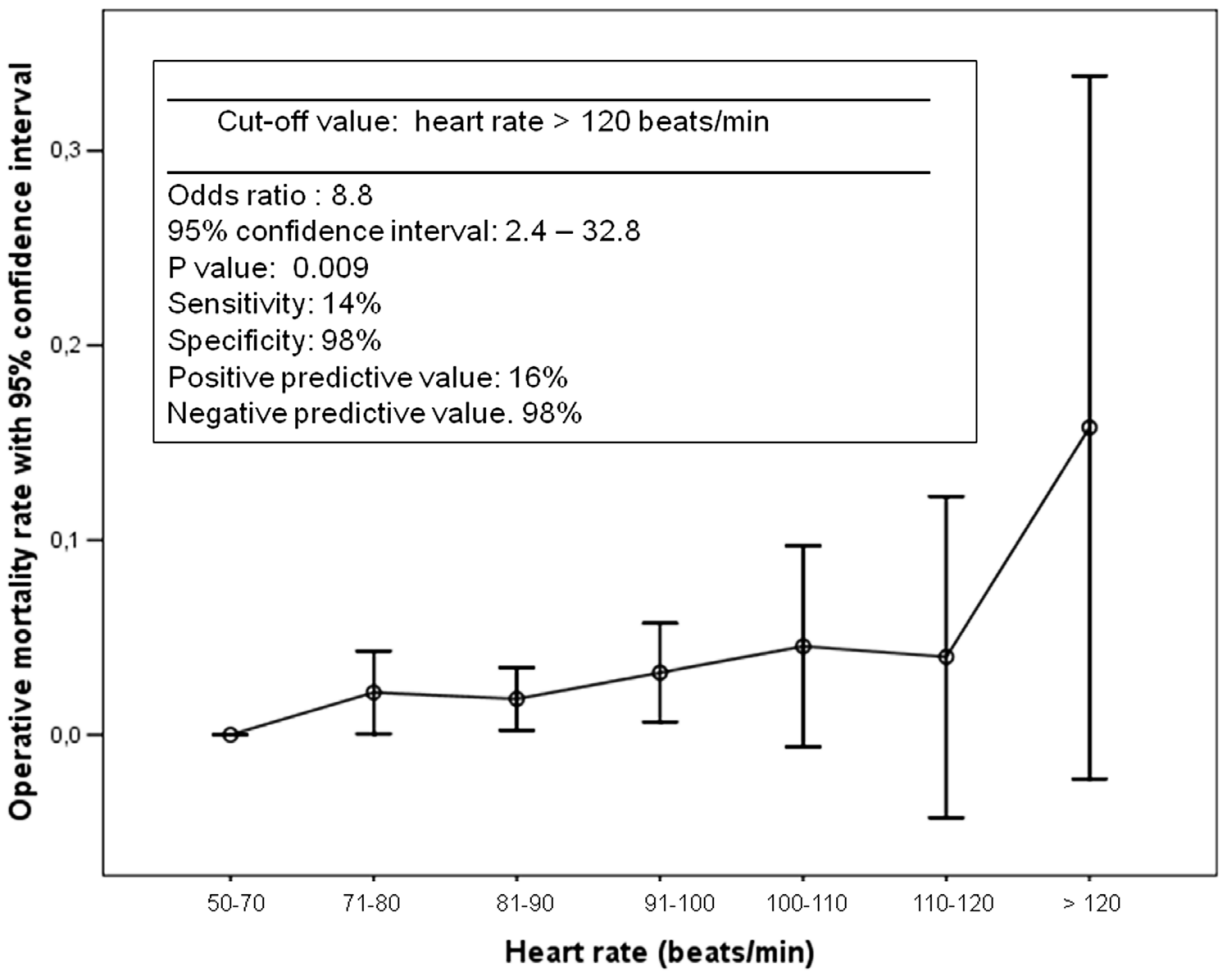
Heart rate and operative mortality. Association between heart rate at the admission in the intensive care unit for relevant categories, with parameters for the identified cut-off value.

Figures 1 and 2 report the results of this analysis. Patients were grouped into relevant categories according to their values of HR and BL. The graphical analysis demonstrated a non-linearity of the relationship, with a significant increase in operative mortality reached for HR values >120 beats per minute and BL values >4 mmol/L. These values had a good specificity and negative predictive value, but a poor sensitivity and positive predictive value. Nineteen (2%) patients had a HR >120 beats per minute (operative mortality rate: 15.8%); 22 (2.4%) patients had a BL value >4 mmol/L (operative mortality rate: 22.7%).

The multivariable logistic regression model was therefore re-fitted using these cut-off values (table 3). This analysis confirmed the significance of both the parameters in determining operative mortality, with an OR of 6.7 for HR >120 beats per minute and 13.4 for BL >4 mmol/L.

Both the logistic EuroSCORE and the ACEF score were tested for accuracy and calibration in predicting operative mortality. The logistic EuroSCORE had a good accuracy with an AUC of 0.85, an acceptable calibration at the Hosmer-Lemeshow statistics (χ^2^: 11.9, P = 0.153), but the predicted mortality rate (7.4%, 95% confidence interval 6.8%–8%). was significantly (P<0.05) larger than observed.

The ACEF score had a good accuracy with an AUC of 0.81, an acceptable calibration at the Hosmer-Lemeshow statistics (χ^2^: 10.9, P = 0.206), and the predicted mortality rate (1.38%, 95% confidence interval 1.33%–1.42%) did not significantly differ from the observed.

**Figure 3:**
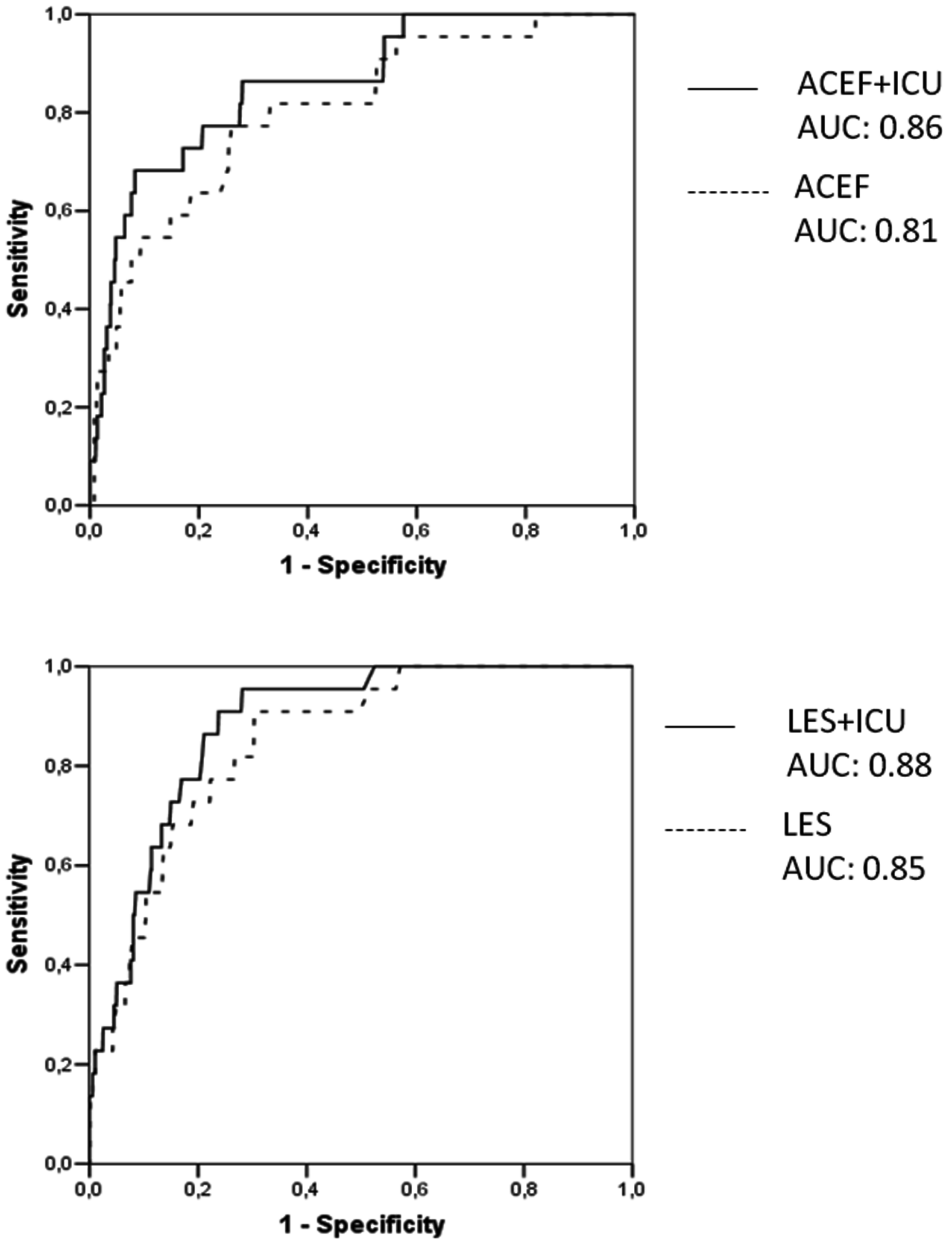
Predictive power of the models. Receiver operating characteristics analysis with area under the curve (AUC) for the logistic EuroSCORE (LES) and the ACEF score, before and after correction for the parameters at the admission in the intensive care unit (ICU).

The weight of the two parameters at the admission in the ICU was investigated for changes in the accuracy of the logistic EuroSCORE and ACEF score. According to the specific OR of the conditions HR >120 beats per minute and BL >4 mmol/L, the two preoperative risk models were re-arranged by including the two parameters at the admission in the ICU (figure 3). The AUC of the logistic EuroSCORE increased to 0.88 and that of the ACEF increased to 0.86, without relevant changes in calibration for both the models.

## Discussion

Stratifying the operative mortality risk in cardiac surgery is still an important issue. The existing models have been all validated, but are still criticized for a number of reasons. Basically, they do perform acceptably in terms of accuracy, but calibration and clinical performance are often unsatisfying. The widely used EuroSCORE, both in its additive and logistic models, carries a well-known overestimation of the operative mortality risk [10]–[16]. The main reason for this bias is probably the outdated development of the model, and a new EuroSCORE development study is presently being performed.

Apart from this, it is likely that intraoperative factors may strongly modify the preoperative risk stratification: changes of the operative planning, poor operative results (incomplete coronary revascularization in CABG surgery; residual valvular defects in valvular repairs), inadequate myocardial protection, accidents, individual reactions to surgery and CPB, hemostatic and coagulation disturbances (thromboembolic events and major bleeding), all these as well as other intraoperative factors may severely increase the operative mortality risk up to higher values than the ones identified by preoperative risk models. Conversely, an uneventful operation with optimal surgery, anesthesia, and CPB management may decrease the operative mortality risk to values lower than the expected.

Our study demonstrates that the accuracy of two preoperative risk scores (ACEF score and logistic EuroSCORE) may be increased by considering two parameters at the admission in the ICU that are nowadays routinely measured (BL and HR).

The association of elevated BL values at the admission in the ICU with hospital/operative mortality was already highlighted in other studies [7], [17]. The same association was found for the peak BL value during CPB [18], [19], that is likely to be associated with the BL value at the admission in the ICU. Different studies proposed different cut-off values, ranging from 2 mmol/L [17] to 3 mmol/L [7], [19] or 4 mmol/L [18]. However, there is consistency in identifying an intraoperative onset (mainly during CPB) of hyperlactatemia as a determinant of bad outcomes, whereas late hyperlactatemia in the ICU is not associated with an increased morbidity and mortality rate [7].

Maillet and coworkers [7] reported an hospital mortality rate of 14.9% in patients with a BL value >3 mmol/L at the admission in the ICU following cardiac operations. Our mortality rate for patients with hyperlactatemia at the admission in the ICU is higher (22.7%) but we used a higher cut-off value (4 mmol/L) and we considered operative and not hospital mortality. Therefore, our study is basically in agreement with the results of Maillet and coworkers.

Hyperlactatemia is a complex condition, which may result from several mechanisms. Tipe A hyperlactatemia is defined as an impaired tissue oxygenation leading to increased anaerobic metabolism and an excessive production of pyruvate (which is then converted to lactate). When the oxygen delivery starts decreasing (due to a decreased cardiac output, extreme hemodilution, or both), the oxygen consumption is maintained until a “critical level” is reached [20]. Below this critical point, the oxygen consumption starts decreasing, becoming dependent on the oxygen delivery, and the failing aerobic energy production is progressively replaced by anaerobic ATP production (pyruvate conversion to lactate). As a result, blood lactate concentration starts raising, and numerous studies have established the use of lactates as a marker of global tissue hypoxia in circulatory shock [21].

Lactate concentration depends on the balance between production and elimination (by the liver). However, the kinetics of lactates clearance depends basically on the production rate, because hepatic clearance appears to be preserved even during cardiogenic shock [22]. Hyperlactatemia is almost invariably associated with hyperglycemia during postoperative cardiogenic shock in cardiac surgery [22], as a consequence of increased concentrations of plasma cortisol and glucagone. Moreover, during severe sepsis and cardiogenic shock insulin resistance may appear, which favours glycolisis and and glucose-lactate cycling [23]. Finally, lactate itself may be converted to glucose through the Cori cycle [23]. As a matter of fact, during CPB there is a direct correlation between peak blood glucose levels and peak blood lactate concentration [19].

Endogenous or exogenous cathecolamines may determine or worsen hyperlactatemia, and experimental studies demonstrated that epinephrine may induce an increase in the lactate plasma concentration to a greater extent than nor-epinephrine, even if this point is still a matter of debate. All togheter, cathecolamines may certainly increase the total oxygen delivery by improving the cardiac output, but often at the expenses of a severe regional maldistribution of flow.

In our study, a BL value >4 mmol/L at the admission in the ICU is strongly associated (OR 13.4) with operative mortality. Our interpretation is that patients presenting with this condition probably suffered a prolonged inadequate oxygen delivery during the intraoperative period, leading to anaerobic energy production. An inadequate oxygen delivery may result from (i) a poor cardiac output, (ii) a poor arterial oxygen content (severe anemia), (iii) increased oxygen demands, and (iiii) a combination of these factors.

Increased oxygen demands are common in other settings like severe sepsis, but difficult to hypothesize in patients narcotized and paralized immediately after the operation, with a normal or moderately decreased core temperature. The hematocrit value at the admission in the ICU was not associated with differences in survival rate, therefore decreasing possibility that severe anemia was a major determinant of the poor oxygen delivery. Therefore, an inadequate cardiac output is likely to be the main source of an inadequate oxygen delivery, leading to hyperlactatemia and ultimately to an increased operative mortality risk.

Blood lactate measurement is an easy and powerful marker of this condition; hyperlactatemia is of course associated with changes in arterial blood gas values (lower pH and HCO_3_^−^, and higher pCO_2_) and offers a combined index of these changes when they depend on the buffering of excess protons production.

The second independent predictor (at the admission in the ICU) of operative mortality is a HR >120 beats per minute, with an O.R. of 6.7. This observation is similar to the one reported by Higgins and coworkers [8], who identified a cut-off value at 100 beats per minute as independent predictor of hospital mortality in a selected population of CABG patients. The higher cut-off value identified in our study is probably justified by the inclusion of valve operations: coronary patients are usually preoperatively treated with drugs limiting the heart rate, that are not routinely used in mitral valve patients and in patients with aortic regurgitation. However, the pathophysiological meaning of tachycardia at the admission in the ICU is similar in both the population, being probably the result of compensatory mechanisms for a decreased stroke volume and/or of the use of inotropic drugs. This, again, stresses the interpretation of a poor cardiac output as a link between heart rate and operative mortality.

There are some limitations in our study. Pulmonary artery catheters were not routinely used, and the derived hemodynamic parameters were not available for this analysis. Central or mixed venous saturation was not available as well in all patients, and we cannot exclude that this parameter may be an independent predictor of operative mortality, being well recognized its validity as a marker of increased peripheral oxygen extraction in presence of an inadequate cardiac output. Blood glucose concentration at the arrival in the ICU was not available in our files, and again we cannot exclude this parameter as an independent predictor of operative mortality. Finally, we did not include parameters related to therapeutic decisions (use of inotropic drugs and/or intra-aortic balloon pump) limiting our analysis to the patient-derived biological signals.

However, hyperlactatemia is strongly intercorraleted with hyperglicemia during cardiogenic shock [22]; cardiogenic shock is treated with cathecolamines; and cathecolamines themselves induce hyperglicemia. Therefore, when mortality is basically due to a low cardiac output state (as happened in 82% of our patients), this is a complex model with several interactions. In this setting, it appears reasonable to use lactate production as a single marker of organ dysoxia predictive for operative mortality.

The existing severity models for ICU patients do not perform well after cardiac operations, and do not increase the accuracy of preoperative risk models for operative mortality [9]. This is probably due to the inclusion of parameters that are strongly affected by the nature of a cardiac operation with CPB. In this environment, leukocytosis is mainly related to the inflammatory effects of CPB; core temperature may be affected by the degree of hypothermia during CPB; and the Glasgow Coma Scale cannot be evaluated at the admission in the ICU. Conversely, our model increased the accuracy of both the logistic EuroSCORE and the ACEF score.

Our results should be interpreted within the complex context of risk stratification and predictive models. It could be questioned whether the relatively small increase in accuracy justifies the use of additional parameters measured at the admission in the ICU. Moreover, the use of ROC models and AUCs for comparing different predictive models or improving the accuracy of a single model is not universally accepted and requires caution [24]. Calibration and discrimination may be more important, in clinical terms, thansimple AUC-determined accuracy. However, it is outside the purposes of the present study to address the complex issue of risk stratification models.

A double-staged operative mortality risk assessment may offer relevant advantages in the cardiac surgery scenario. The detection of hyperlactatemia and/or tachycardia at the admission in the ICU provides a signal that the patient is in need for specific care and observation during the postoperative course. This may suggest the application of an extensive hemodynamic monitoring (with pulmonary artery catheters and/or transesophageal echocardiography) to identify the nature and quantify the severity of a possible heart failure. Finally, within an institutional approach aimed to assess the quality of the surgical care, a predicted operative mortality rate at the admission in the ICU higher than the one preoperatively predicted is suggestive for an intraoperative management that deserves improvements.
